# Predicting heart failure in asymptomatic diabetes: derivation and internal validation of a clinical prediction model for early detection of diabetic cardiomyopathy

**DOI:** 10.3389/fendo.2026.1882872

**Published:** 2026-06-22

**Authors:** Yu Cao, Wenwen Chen, Yunyuan Tian, Yao Li, Lu Xu, Haifeng Tang

**Affiliations:** 1 Department of Chinese Materia Medica and Natural Medicines, School of Pharmacy, The Air Force Medical University, Xi’an, Shaanxi, China; 2 The College of Pharmacy, Shaanxi University of Chinese Medicine, Xianyang, China; 3 The College of Life Pharmacy, Northwest University, Xi’an, Shaanxi, China

**Keywords:** diabetic cardiomyopathy, diastolic function, global longitudinal strain, heart failure, left atrial reservoir strain, prediction model, subclinical cardiac dysfunction, type 2 diabetes mellitus

## Abstract

**Objective:**

To develop and internally validate a clinical prediction model for identifying asymptomatic type 2 diabetes (T2DM) patients at risk of incident heart failure (HF) or progression of subclinical cardiac dysfunction.

**Methods:**

This single-center retrospective study included 326 asymptomatic T2DM patients with preserved LVEF, all with ≥3 echocardiographic assessments over 24 months. The primary outcome was a composite of incident clinical HF (classified as HFpEF or HFrEF based on symptoms, hospitalization, natriuretic peptides, and follow-up echo) and imaging-defined progression of subclinical dysfunction. Candidate predictors (clinical variables, GLS, LASr, E/e′, LAVI, LVMI, NT-proBNP, galectin-3) were pre-specified. Missing data were handled by multiple imputation. A risk score was derived using multivariable Cox regression with Fine-Gray competing risk analysis, and optimism was assessed via bootstrap, 5-fold cross-validation, and internal split-sample validation.

**Results:**

Over 24 months, LV GLS modestly declined (17.4 ± 2.0% to 16.9 ± 2.1%), while LASr deteriorated more (38.5 ± 7.2% to 34.0 ± 6.5%). Independent predictors of the composite outcome were LASr ≤24% (HR 3.58), NT-proBNP ≥120 pg/mL (HR 2.48), galectin-3 ≥15 ng/mL (HR 1.79), age ≥70 years, diabetes duration ≥12 years, BMI ≥30 kg/m², and UACR ≥60 mg/g; SGLT2i/GLP-1 RA use was associated with lower observed risk. During follow-up, 40 patients reached the composite outcome (18 clinical HF: 14 HFpEF, 4 HFrEF; 22 imaging-only progression). The scoring system stratified patients into low- (≤3 points), intermediate- (4–6), and high-risk (≥7) groups, with 24-month cumulative incidences of 4.2%, 11.7%, and 27.5% (Gray’s test P<0.001). The full model had a C-statistic of 0.835 (bootstrap-corrected 0.828) and good calibration (Brier score 0.068). Adding LASr and galectin-3 improved discrimination (ΔC=0.058 and 0.012, both P<0.05).

**Conclusion:**

Integrating LASr, galectin-3, and clinical risk factors may help identify asymptomatic T2DM patients at higher risk of incident HF or subclinical progression. This internally validated model may support preliminary risk stratification for suspected early diabetic cardiomyopathy, but external validation and clinical utility assessment are needed before routine use.

## 1 Introduction

Type 2 diabetes mellitus (T2DM) is among the most significant chronic diseases globally. The 2024 IDF Diabetes Atlas reports 589 million affected adults (11.11%), projected to reach 853 million by 2050, intensifying the burden of diabetes-related organ damage (1). Among cardiovascular complications, heart failure (HF) represents a clinically burdensome and often underestimated outcome. Meta-analyses demonstrate that T2DM patients have a significantly higher risk of developing HF, persisting across various baseline cardiovascular statuses (2). Real-world studies indicate that diabetic cardiomyopathy (DCM), as a distinct pathological entity, has a non-negligible prevalence in T2DM, accounting for approximately 2.9% of the overall population and reaching 16.2% among those with asymptomatic Stage B HF (3). Diabetes and HF form a mutually reinforcing vicious cycle, increasing risk and worsening prognosis, thus constituting a critical area for intervention (4).

DCM is generally regarded as a diabetes-related myocardial phenotype characterized by structural remodeling and functional impairment that occur in the absence of overt coronary artery disease, significant valvular disease, or other primary cardiomyopathies. Metabolic dysregulation, particularly altered glucose and lipid substrate utilization, is considered a key mechanism underlying early diabetic myocardial injury (5). Persistent hyperglycemia and insulin resistance may further promote oxidative stress, inflammatory activation, and myocardial fibrosis, thereby contributing to progressive structural and functional remodeling (6). Inflammatory signaling, including macrophage-mediated pathways, may provide a biological link between metabolic stress and myocardial tissue injury (7). Mitochondrial dysfunction may also contribute to the transition from metabolic abnormalities to impaired myocardial mechanics (8). The diabetic heart is characterized by enhanced fatty acid but restricted glucose utilization, accompanied by abnormal ketone and branched-chain amino acid metabolism, increasing oxygen consumption and reducing energy efficiency (9). Furthermore, diabetes-related HF with preserved ejection fraction (HFpEF) is now characterized as a multi-organ interaction disease, where adipose tissue, liver, gut, and immune system engage in systemic crosstalk with the heart via metabolic and inflammatory signals, accelerating fibrosis and diastolic dysfunction (10).

Clinically, DCM often begins subclinically, characterized by early myocardial electrical vulnerability including prolonged action potential duration, altered calcium and potassium channel activity, and increased susceptibility to arrhythmias. Early manifestations include impaired LV diastolic function, LA enlargement, and abnormal myocardial mechanics, even when LVEF remains normal (11). Therefore, LV GLS based on speckle-tracking echocardiography, along with diastolic burden and LA function indicators, not only provide sensitive measures of mechanical function but may also serve as indirect markers of myocardial electromechanical stability and arrhythmia susceptibility. A meta-analysis focusing on HFpEF showed that impaired GLS, reduced LA reservoir strain, and elevated E/e′ independently predict cardiovascular death or HF hospitalization (12). A recent review also indicates that LA strain abnormalities are closely associated with atrial fibrillation, HF hospitalization, and death in T2DM (13). Regarding treatment, although novel glucose-lowering drugs have shown cardiac benefits—randomized studies indicate that liraglutide, empagliflozin, or their combination can improve LA strain (14), and SGLT2 inhibitors can improve E/e′, LA volume index (LAVI), and GLS in T2DM patients with HF with mildly reduced ejection fraction (HFmrEF) (15)—even the latest trials like STEP-HFpEF DM focus primarily on syndrome-level benefits rather than DCM-specific remodeling and long-term functional trajectories (16).

Against this backdrop, the present study used a longitudinal clinical cohort to systematically evaluate cardiac functional trajectories and to develop a prediction model for identifying asymptomatic T2DM patients at risk of incident HF or progression of subclinical cardiac dysfunction, focusing on systolic and diastolic function, structural remodeling, and clinical outcomes. The model is intended to provide a preliminary framework for risk stratification of asymptomatic T2DM patients in tertiary care settings, pending further external validation.

## 2 Materials and methods

### 2.1 Study design

This study was a single-center, retrospective longitudinal cohort investigation aimed at developing and internally validating a clinical prediction model for incident HF or progression of subclinical cardiac dysfunction in asymptomatic patients with T2DM. In this study, suspected early DCM was operationally defined as diabetes-related subclinical myocardial abnormality in patients without overt coronary artery disease, significant valvular heart disease, persistent severe arrhythmia, non-diabetic cardiomyopathy, or end-stage systemic disease. The study population was identified from the electronic medical record system, cardiac imaging database, and standardized follow-up registry, representing adult T2DM patients receiving routine care at a tertiary general hospital in Xi’an, China.

The overall study period spanned January 1, 2023, to December 31, 2025, with patient enrollment occurring between January 1, 2023, and December 31, 2024. Clinical and imaging follow-up data were collected until December 31, 2025, providing a continuous two-year longitudinal observation window.

A stratified, stepwise selection approach was applied. Adult patients diagnosed with type 2 diabetes mellitus during the study period were first identified through the hospital information system. Patients with baseline myocardial structural or functional abnormalities were then screened using the cardiac imaging database to capture diabetes-related myocardial changes. Only patients with continuous cardiac ultrasound records, comprising at least three examinations—baseline, approximately 12-month, and approximately 24-month follow-ups—were included to ensure reliable trajectory modeling and longitudinal analysis.

From this initial screening, 512 patients met the basic inclusion criteria. Patients with >30% missing key variables were excluded. For the remaining patients, missing data were imputed using multiple imputation by chained equations (MICE) with 20 imputed datasets. The imputation model included all candidate predictors, the composite outcome indicator, and follow-up time to preserve associations between predictors and outcomes. The proportion of missingness for individual variables was below 15%, with the highest missingness observed for galectin-3 (12.6%), NT-proBNP (9.8%), and LASr (8.3%). Convergence was assessed by visual inspection of trace plots and distributional comparisons between observed and imputed values. Reasons for exclusion included: severe coronary artery stenosis or prior myocardial infarction (n=58), moderate or greater valvular heart disease (n=32), persistent atrial fibrillation or other severe arrhythmias (n=26), non-diabetic cardiomyopathy (n=21), end-stage renal disease or dialysis (n=14), active malignancy (n=9), and incomplete follow-up or >30% missing key data (n=26).

This retrospective study was approved by the Medical Ethics Committee of The First Affiliated Hospital of Air Force Medical University (approval No. 20231250). Written informed consent was waived because all data were de-identified and no additional interventions were performed.

### 2.2 Inclusion and exclusion criteria

Eligible participants were adults (≥18 years) with a confirmed diagnosis of type 2 diabetes mellitus, documented either in the electronic medical records or by a specialist physician during the study period. Inclusion further required objective evidence of subclinical myocardial structural or functional abnormality at baseline, defined by at least one of the following echocardiographic thresholds: left ventricular diastolic dysfunction (E/e′ >14 or septal e′ <7 cm/s), left ventricular hypertrophy (LV mass index ≥115 g/m² in males or ≥95 g/m² in females), left atrial enlargement (LAVI >34 mL/m²), mildly reduced LVEF (LVEF 50%–54%), or abnormal global longitudinal strain (absolute GLS <16%). Only patients with longitudinal cardiac imaging data, defined as at least three echocardiograms across the observation period, were considered to ensure reliable trajectory assessment.

Exclusion criteria were designed to eliminate confounding cardiac conditions or systemic factors that could independently affect myocardial structure or function. Patients with prior ischemic heart disease, significant valvular disease, congenital or genetic cardiomyopathy, myocardial injury from non-diabetic causes (e.g., myocarditis, alcoholic or drug-induced cardiomyopathy, peripartum cardiomyopathy), persistent arrhythmias, end-stage renal disease, active malignancy, or those undergoing major cardiac interventions were excluded. Additionally, patients with incomplete follow-up or missing core study variables (>30%) were removed to maintain the robustness of longitudinal analyses. Because these abnormalities may overlap with hypertensive heart disease, obesity-related remodeling, aging-related myocardial changes, or occult ischemic disease, the study cohort should be interpreted as a high-risk asymptomatic T2DM population with subclinical cardiac abnormalities.

All included patients fulfilled the ACC/AHA Stage B HF criteria, defined as asymptomatic structural or functional cardiac abnormalities (e.g., left ventricular hypertrophy, diastolic dysfunction, left atrial enlargement, or abnormal global longitudinal strain) in the absence of clinical heart failure symptoms.

### 2.3 Study methods

#### 2.3.1 Clinical and laboratory data collection

All relevant clinical variables were extracted from the hospital electronic medical record system using a standardized data collection template. Baseline demographic information included age, sex, body mass index (BMI), and diabetes duration. Laboratory parameters included glycated hemoglobin (HbA1c), fasting plasma glucose, lipid profile, serum creatinine, and estimated glomerular filtration rate (eGFR). Comorbidities such as hypertension, obesity, and chronic kidney disease were identified based on definitive clinical diagnoses, supported by at least two medical records. Medication use at baseline was recorded to reflect stable treatment status, focusing on glucose-lowering and cardiovascular-related therapies.

#### 2.3.2 Cardiac imaging assessment

All included patients underwent standardized transthoracic echocardiography using the same ultrasound system (GE Vivid E95) with vendor-specific speckle-tracking software (EchoPAC), and image acquisition was performed by qualified sonographers according to American Society of Echocardiography guidelines. All analyses were performed on the same software platform to ensure consistency. Left ventricular ejection fraction (LVEF) was calculated using the biplane Simpson’s method. Diastolic function parameters included early diastolic mitral inflow velocity (E), early diastolic mitral annular velocity (e’) via tissue Doppler, and the E/e’ ratio. LAVI and left ventricular mass index (LVMI) were measured and indexed to body surface area. Global longitudinal strain (GLS) was assessed using two-dimensional speckle-tracking echocardiography. To minimize measurement error and observer bias, imaging data were independently analyzed by two experienced physicians blinded to clinical information, with a third senior physician resolving discrepancies. To assess echocardiographic reproducibility, 40 randomly selected examinations were re-analyzed. Inter-observer variability was assessed by comparing measurements obtained independently by the two physicians. Intra-observer variability was assessed by repeated analysis by the same physician after a 4-week interval, blinded to the initial results. Reproducibility of key strain parameters, including LV GLS and LASr, was evaluated using intraclass correlation coefficients (ICCs) and Bland–Altman analyses.

#### 2.3.3 Follow-up methods

Baseline was defined as the time when patients first met the inclusion criteria and completed a full clinical and imaging assessment. Follow-up was pre-specified at approximately 12- and 24-months post-baseline. The 12-month follow-up was defined as 12 months ± 3 months, and the 24-month follow-up as 24 months ± 3 months. The closest high-quality echocardiographic examination within each window was selected. All follow-up data were electronically extracted and verified to ensure continuity and completeness, forming a robust longitudinal dataset for model development and validation.

#### 2.3.4 Outcome definition and model development

The primary outcome was a composite of incident clinical HF and imaging-defined progression of subclinical cardiac dysfunction. Incident clinical HF was defined as new-onset symptomatic HF documented in clinical records or HF-related hospitalization, supported by elevated natriuretic peptide levels and follow-up echocardiographic evidence of cardiac dysfunction. Clinical HF events were further classified as HFpEF when LVEF was ≥50% with evidence of elevated filling pressure or structural heart disease, and as HFrEF when LVEF was <40% during follow-up. Patients with LVEF between 40% and 49% were reviewed separately; because of the small number of such cases, they were not analyzed as an independent subgroup.

Imaging-defined progression of subclinical cardiac dysfunction was defined as worsening cardiac structure or function on follow-up echocardiography without documented HF symptoms or HF hospitalization. Progression required at least two of the following: relative GLS deterioration ≥15% from baseline or absolute GLS <16%; LASr ≤24% or relative LASr decline ≥20%; E/e′ >14; LAVI >34 mL/m² with further increase from baseline; or LVMI increase consistent with progressive remodeling. When both clinical HF and imaging progression occurred in the same patient, the event was classified as clinical HF. Outcome events were adjudicated by two experienced cardiologists blinded to baseline clinical variables, with disagreements resolved by a third senior cardiologist.

A total of 26 candidate predictors were pre-specified before model fitting based on clinical relevance, prior literature, and biological plausibility. These included demographic variables, metabolic factors, comorbidities, medication use, biomarkers, and echocardiographic indices. To reduce model overfitting given the limited number of outcome events, variables with strong collinearity were not entered simultaneously, and candidate selection was guided by both clinical relevance and univariable associations. Variables with P < 0.10 in univariable analysis were considered for multivariable modeling, but final model retention also required clinical interpretability and stability across bootstrap resampling. Multivariable Cox proportional hazards regression was used to derive the prediction model, and Fine–Gray competing risk analysis was performed as a sensitivity analysis accounting for non-cardiovascular death. Internal validation was performed using bootstrap resampling, 5-fold cross-validation, and internal split-sample validation.

### 2.4 Outcome measures

#### 2.4.1 Primary outcome measures

Left Ventricular GLS Trajectory.

Assessed using 2D speckle-tracking echocardiography, averaged across 18 segments from standard apical views. Absolute GLS values were used, with lower values indicating impaired systolic function. GLS change over 24 months and rate of change between time points were calculated to reflect longitudinal systolic function dynamics.

Left Ventricular Filling Pressure Index (E/e’ Ratio) Trajectory.

Calculated from early diastolic mitral inflow velocity (E) and annular velocity (e’). This index reflects diastolic function and left ventricular filling pressure, serving as an indirect marker of electromechanical coupling. Values were tracked longitudinally.

Myocardial Mechanical Efficiency Index (MMEI = |GLS|/E/e’).

An exploratory composite integrating systolic and diastolic performance to assess myocardial mechanical efficiency.

Structural-Functional Coupling Index (SFCI = LVMI/|GLS|).

An exploratory index reflecting the relationship between myocardial mass and systolic performance.

#### 2.4.2 Secondary outcome measures

LVEF Trajectory – global systolic function.

LAVI Trajectory – chronic diastolic load.

LVMI Trajectory – structural remodeling.

NT-proBNP Trend – biomarker of ventricular wall stress.

Rate of Myocardial Functional Decline (ΔGLS/time) – quantitative measure of systolic deterioration.

Diastolic Burden Index (DBI = E/e’ × LAVI) – exploratory index of cumulative diastolic load.

Clinical Outcome Events – new-onset symptomatic HF or HF hospitalization, further classified as HFpEF, HFrEF, or HF with mildly reduced ejection fraction when applicable. Imaging-only progression of subclinical cardiac dysfunction was recorded separately from clinical HF events.

### 2.5 Core predictive variables and statistical methods

#### 2.5.1 Core predictive variables

The primary predictors for the development of heart failure in asymptomatic diabetes included:

GLS and its rate of change – sensitive early marker of systolic function.

E/e’ ratio and DBI (Diastolic Burden Index) – indicators of diastolic function and myocardial load.

LVMI and LAVI – measures of structural remodeling and chronic pressure load.

MMEI and SFCI – exploratory indices reflecting structure-function coupling.

NT-proBNP – biomarker of heart failure risk.

Baseline clinical features – age, sex, BMI, diabetes duration, comorbidities, and medications.

Note: MMEI, SFCI, and DBI are exploratory indicators; the core model should prioritize GLS, E/e’, LVMI, LAVI, NT-proBNP, and relevant clinical variables.

#### 2.5.2 Statistical methods

All analyses were performed using R software. Continuous variables were expressed as mean ± SD or median (IQR) depending on distribution; categorical variables as frequencies (percentages). Normality was assessed using the Shapiro–Wilk test. Between-group comparisons were performed using ANOVA or Kruskal–Wallis tests for continuous variables and chi-square or Fisher’s exact tests for categorical variables.

Longitudinal changes in echocardiographic indices were analyzed using linear mixed-effects models to account for repeated measurements within individuals. Time was modeled as a categorical variable (baseline, 12, and 24 months), with patient-specific random intercepts. Fixed effects included time, and adjusted models further included age, sex, BMI, diabetes duration, systolic blood pressure, eGFR, HbA1c, hypertension, CKD, and cardiovascular-related medications. Least-squares mean differences from baseline, 95% confidence intervals (CIs), and P values were reported for each follow-up time point.

Echocardiographic reproducibility was assessed in 40 randomly selected examinations. Inter- and intra-observer variability for key parameters (LV GLS, LASr) were evaluated using intraclass correlation coefficients (ICCs, two-way random-effects, absolute agreement) and Bland–Altman plots. ICC >0.75 was considered good, >0.90 excellent.

For prediction model development, 26 pre-specified candidate variables were screened using univariable Cox regression (P < 0.10), with final multivariable model selection based on statistical significance, clinical relevance, collinearity assessment (variance inflation factors), and bootstrap selection stability. Backward elimination was used only as an exploratory step. Missing data were handled by multiple imputation via chained equations (20 datasets, missing-at-random assumption), with estimates pooled using Rubin’s rules. Sensitivity analyses included complete-case analysis.

Internal validation included 1,000 bootstrap resamples, 5-fold cross-validation, and internal split-sample validation (70% derivation, 30% validation). Model performance was evaluated using the C-statistic, calibration slope, calibration intercept, and Brier score. No external validation cohort was available.

Hierarchical adjustment models were constructed: Model 1 adjusted for age and sex; Model 2 additionally included BMI, diabetes duration, HbA1c, systolic blood pressure, and eGFR; Model 3 further included cardiovascular-related medications. All covariates were pre-specified based on clinical relevance.

Additional analyses included latent class growth analysis (LCGA) to identify distinct cardiac function trajectories using Bayesian Information Criterion and model stability for class selection. Patients were assigned to trajectory groups by maximum posterior probability, and associations with baseline clinical features were assessed via multinomial logistic regression. Individual-level slopes of myocardial function decline and composite indices were calculated. Sensitivity analyses included exclusion of patients undergoing major cardiovascular interventions, repeating analyses within medication subgroups, and using alternative time window definitions. All tests were two-sided with P < 0.05 considered statistically significant, and Bonferroni corrections were applied for multiple comparisons in supplementary analyses.

## 3 Results

### 3.1 Patient selection and baseline characteristics

**Figure 1:**
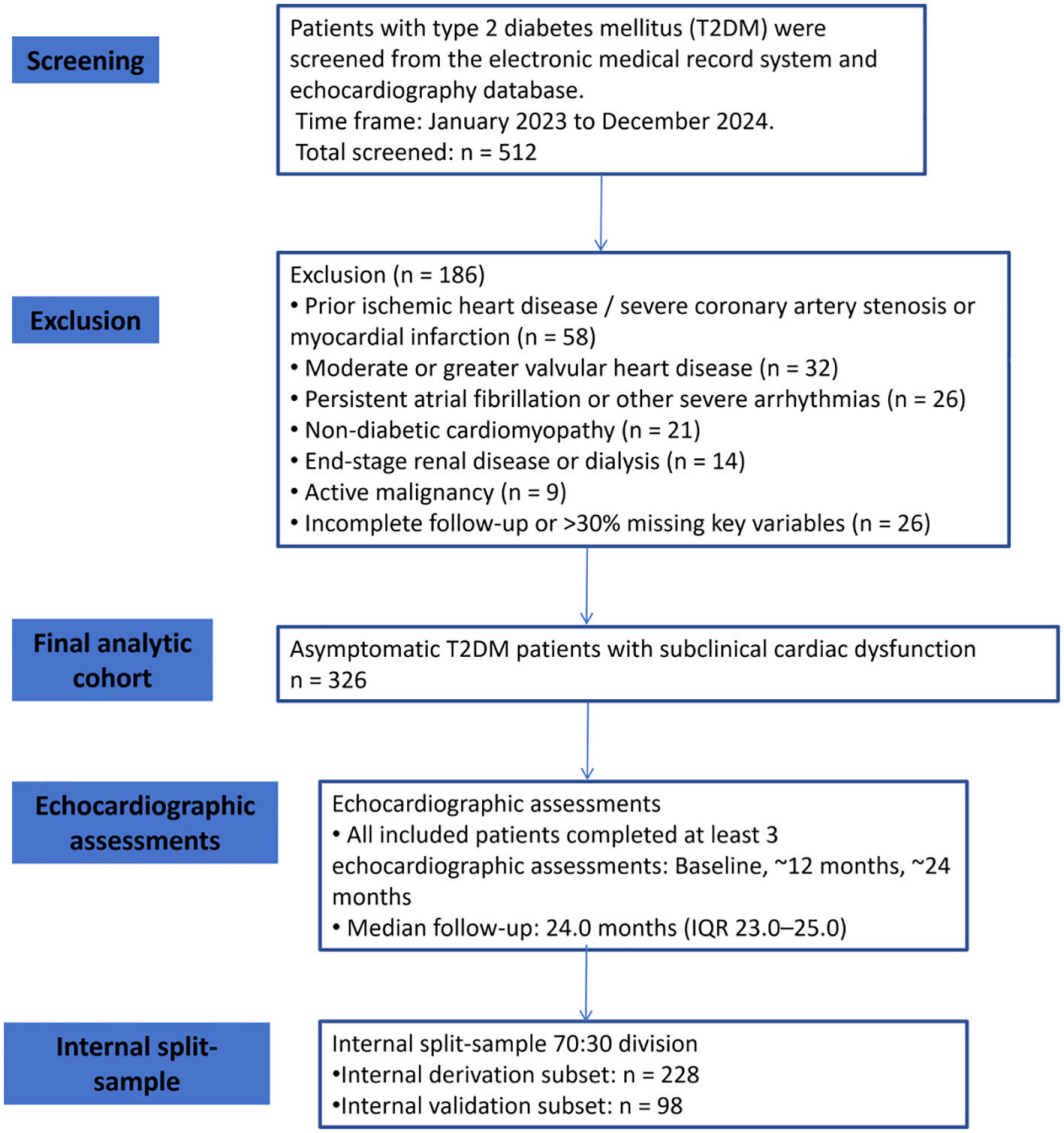
Study flowchart. T2DM, type 2 diabetes mellitus; IQR, interquartile range. The 70:30 derivation-validation split was used for internal split-sample validation and does not represent external validation.

Of 512 T2DM patients screened, 326 were included (**Figure 1**). Baseline characteristics (**Table 1**) included mean age 61.3 ± 9.4 years, 55.8% male, diabetes duration 9.0 years, HbA1c 7.8 ± 1.1%, LVEF 57.1 ± 5.7%, GLS 17.4 ± 2.0%, E/e′ 11.3 ± 2.6, LAVI 35.0 ± 6.5 mL/m², and LVMI 103.0 ± 17.4 g/m². Over 24 months, 40 patients experienced the composite outcome versus 286 event-free (**Table 1A**). Event patients showed older age, longer diabetes duration, higher BMI, more hypertension/CKD, higher NT-proBNP and galectin-3, worse LASr/GLS, and higher E/e′/LAVI, with no differences in sex, smoking, or lipid-lowering therapy.

**Table 1:** Baseline characteristics of the study population (n=326).

Characteristic	Overall (n=326)
Age, years	61.3 ± 9.4
Male sex, n (%)	182 (55.8)
Diabetes duration, years	9.0 (6–13)
BMI, kg/m²	26.5 ± 3.3
HbA1c, %	7.8 ± 1.1
1,5-AG, μg/mL	13.5 (10–17)
UACR, mg/g	22 (12–38)
eGFR, mL/min/1.73 m²	85 ± 18
NT-proBNP, pg/mL	95 (60–140)
hs-TnI, ng/L	4.1 (2.7–6.0)
Galectin-3, ng/mL	12.0 ± 3.5
hs-CRP, mg/L	1.8 (1.0–3.5)
LVEF, %	57.1 ± 5.7
LV GLS, % (absolute)	17.4 ± 2.0
LASr, %	38.5 ± 7.2
LAScd, %	22.0 ± 4.8
LASct, %	16.5 ± 4.0
LASr/LAScd ratio	1.75 ± 0.35
E/e′ ratio	11.3 ± 2.6
LAVI, mL/m²	35.0 ± 6.5
LV mass index, g/m²	103.0 ± 17.4
Hypertension, n (%)	201 (61.7)
CKD stage, n (%)	
No CKD	266 (81.6)
CKD stage 1–2	38 (11.7)
CKD stage 3	18 (5.5)
CKD stage 4–5	4 (1.2)
Current smoker, n (%)	72 (22.1)
Former smoker, n (%)	49 (15.0)
Never smoker, n (%)	205 (62.9)
Lipid-lowering therapy, n (%)	
No lipid-lowering therapy	96 (29.4)
Low/moderate-intensity statin	170 (52.1)
High-intensity statin or statin plus ezetimibe/PCSK9 inhibitor	60 (18.4)
SGLT2i/GLP-1 RA use, n (%)	107 (32.8)
ACEi/ARB use, n (%)	208 (63.8)
Statin use, n (%)	230 (70.6)

**Table 1A:** Baseline characteristics according to composite outcome status.

Characteristic	Event group (n=40)	Non-event group (n=286)	P value
Age, years	68.6 ± 7.8	60.3 ± 9.2	<0.001
Male sex, n (%)	24 (60.0)	158 (55.2)	0.57
Diabetes duration, years	13.0 (10–16)	8.0 (6–12)	<0.001
BMI, kg/m²	28.5 ± 3.6	26.2 ± 3.1	<0.001
HbA1c, %	8.2 ± 1.2	7.7 ± 1.1	0.01
Hypertension, n (%)	31 (77.5)	170 (59.4)	0.03
CKD stage, n (%)			0.04
No CKD	27 (67.5)	239 (83.6)	
CKD stage 1–2	7 (17.5)	31 (10.8)	
CKD stage 3	5 (12.5)	13 (4.5)	
CKD stage 4–5	1 (2.5)	3 (1.0)	
UACR, mg/g	54 (28–86)	20 (11–35)	<0.001
eGFR, mL/min/1.73 m²	76 ± 19	86 ± 17	0.002
NT-proBNP, pg/mL	150 (105–215)	88 (58–128)	<0.001
Galectin-3, ng/mL	15.2 ± 3.8	11.6 ± 3.1	<0.001
LVEF, %	55.8 ± 5.9	57.3 ± 5.6	0.11
LV GLS, % absolute	16.2 ± 2.1	17.6 ± 1.9	<0.001
LASr, %	27.8 ± 6.4	40.0 ± 6.0	<0.001
E/e′ ratio	13.5 ± 2.9	11.0 ± 2.4	<0.001
LAVI, mL/m²	40.2 ± 7.1	34.3 ± 6.0	<0.001
LV mass index, g/m²	113.0 ± 19.2	101.6 ± 16.4	<0.001
Current smoker, n (%)	10 (25.0)	62 (21.7)	0.21
Former smoker, n (%)	8 (20.0)	41 (14.3)	
Never smoker, n (%)	22 (55.0)	183 (64.0)	
Lipid-lowering therapy, n (%)			0.48
No lipid-lowering therapy	10 (25.0)	86 (30.1)	
Low/moderate-intensity statin	20 (50.0)	150 (52.4)	
High-intensity statin or statin plus ezetimibe/PCSK9 inhibitor	10 (25.0)	50 (17.5)	
SGLT2i/GLP-1 RA use, n (%)	8 (20.0)	99 (34.6)	0.07
ACEi/ARB use, n (%)	29 (72.5)	179 (62.6)	0.23
Statin use, n (%)	30 (75.0)	200 (69.9)	0.51

Values are presented as mean ± SD, median (IQR), or n (%). The event group included patients with incident clinical HF or imaging-defined progression of subclinical cardiac dysfunction. P values were calculated using Student’s t-test, Mann–Whitney U test, χ² test, or Fisher’s exact test, as appropriate.

### 3.2 Longitudinal trajectories of cardiac function

Over a median follow-up of 24 months, cardiac indices showed modest but significant longitudinal changes (**Table 2**). Linear mixed-effects models revealed that LV GLS declined from 17.4 ± 2.0% to 16.9 ± 2.1% (adjusted mean change: −0.51%, 95% CI −0.70 to −0.32, P<0.001), E/e′ increased from 11.3 ± 2.6 to 12.0 ± 2.8 (change: 0.72, 95% CI 0.46–0.98, P<0.001), and LAVI increased from 35.0 ± 6.5 to 36.2 ± 6.8 mL/m² (change: 1.18, 95% CI 0.70–1.66, P<0.001). Left atrial reservoir strain showed more pronounced deterioration: LASr decreased from 38.5 ± 7.2% to 34.0 ± 6.5% (change: −4.48%, 95% CI −5.12 to −3.84, P<0.001). Reproducibility was good-to-excellent for LV GLS (inter-observer ICC 0.91, intra-observer ICC 0.94) and LASr (0.88 and 0.92, respectively; **Table 3**).

**Table 2:** Longitudinal changes in echocardiographic parameters over 24 months.

Parameter	Baseline	12 months	24 months	Adjusted change at 12 months	P value	Adjusted change at 24 months	P value
LV GLS, % absolute	17.4 ± 2.0	17.1 ± 2.0	16.9 ± 2.1	−0.28 (−0.43 to −0.13)	<0.001	−0.51 (−0.70 to −0.32)	<0.001
E/e′ ratio	11.3 ± 2.6	11.7 ± 2.7	12.0 ± 2.8	0.38 (0.19 to 0.57)	<0.001	0.72 (0.46 to 0.98)	<0.001
LAVI, mL/m²	35.0 ± 6.5	35.6 ± 6.6	36.2 ± 6.8	0.58 (0.22 to 0.94)	0.002	1.18 (0.70 to 1.66)	<0.001
LV mass index, g/m²	103.0 ± 17.4	104.1 ± 17.8	105.0 ± 18.2	1.05 (0.24 to 1.86)	0.011	1.94 (0.88 to 3.00)	<0.001
LASr, %	38.5 ± 7.2	36.1 ± 6.8	34.0 ± 6.5	−2.34 (−2.86 to −1.82)	<0.001	−4.48 (−5.12 to −3.84)	<0.001
LAScd, %	22.0 ± 4.8	21.2 ± 4.7	20.5 ± 4.6	−0.76 (−1.09 to −0.43)	<0.001	−1.45 (−1.86 to −1.04)	<0.001
LASct, %	16.5 ± 4.0	16.1 ± 4.0	15.8 ± 3.9	−0.36 (−0.61 to −0.11)	0.005	−0.68 (−0.99 to −0.37)	<0.001

Values are presented as mean ± SD or adjusted mean change from baseline (95% CI). Adjusted changes were estimated using linear mixed-effects models with patient-specific random intercepts. Models were adjusted for age, sex, BMI, diabetes duration, systolic blood pressure, eGFR, HbA1c, hypertension, CKD, and cardiovascular-related medication use. GLS values are expressed as absolute values; negative changes indicate worsening systolic deformation.

**Table 3:** Reproducibility of key echocardiographic strain parameters.

Parameter	Inter-observer ICC (95% CI)	Inter-observer mean bias	Inter-observer 95% limits of agreement	Intra-observer ICC (95% CI)	Intra-observer mean bias	Intra-observer 95% limits of agreement
LV GLS, % absolute	0.91 (0.85–0.95)	−0.18%	−1.42 to 1.06%	0.94 (0.89–0.97)	−0.10%	−1.05 to 0.85%
LASr, %	0.88 (0.80–0.93)	−0.62%	−4.10 to 2.86%	0.92 (0.86–0.96)	−0.36%	−3.20 to 2.48%
E/e′ ratio	0.86 (0.77–0.92)	0.21	−1.48 to 1.90	0.90 (0.83–0.94)	0.12	−1.22 to 1.46
LAVI, mL/m²	0.89 (0.82–0.94)	−0.74	−4.90 to 3.42	0.93 (0.88–0.96)	−0.42	−3.70 to 2.86

Reproducibility was assessed in 40 randomly selected echocardiographic examinations. ICCs were calculated using a two-way random-effects model with absolute agreement. Bland–Altman mean bias was calculated as observer 1 minus observer 2 for inter-observer variability and repeat measurement minus initial measurement for intra-observer variability.

### 3.3 Predictor selection and multivariable analysis

Among 326 patients, 40 reached the composite outcome. Of 26 pre-specified candidate variables, 13 with P < 0.10 in univariable analysis were considered. Given the limited event number, multivariable modeling was performed cautiously using backward elimination as an exploratory step, with final predictor retention based on significance, clinical relevance, collinearity, and bootstrap stability. Eight predictors were retained, and a Fine–Gray competing risk model was applied (**Table 4**).

**Table 4:** Final predictors and risk scoring for the composite outcome in asymptomatic T2DM.

Predictor (cut-off/unit)	β coefficient	HR (95% CI)	P value	Subdistribution HR	Points assigned	Notes
Age ≥70 years	0.75	2.12 (1.51–2.98)	<0.001	2.08 (1.47–2.94)	2	Clinical risk factor
Diabetes duration ≥12 years	0.6	1.82 (1.27–2.61)	0.001	1.79 (1.24–2.58)	1	Clinical risk factor
BMI ≥30 kg/m²	0.55	1.73 (1.22–2.46)	0.002	1.71 (1.20–2.44)	1	Clinical risk factor
UACR ≥60 mg/g	0.67	1.95 (1.38–2.76)	<0.001	1.92 (1.35–2.73)	2	Marker of renal involvement
NT-proBNP ≥120 pg/mL	0.91	2.48 (1.74–3.54)	<0.001	2.51 (1.75–3.60)	3	Ventricular wall stress
LASr ≤24%	1.28	3.58 (2.25–5.71)	<0.001	3.64 (2.52–5.26)	4	Primary atrial strain marker
Galectin-3 ≥15 ng/mL	0.58	1.79 (1.25–2.56)	0.002	1.76 (1.22–2.54)	2	Fibrotic biomarker
SGLT2i/GLP-1 RA use	–0.72	0.49 (0.35–0.69)	<0.001	0.47 (0.33–0.67)	–1	Inverse observational association

**Figure 2:**
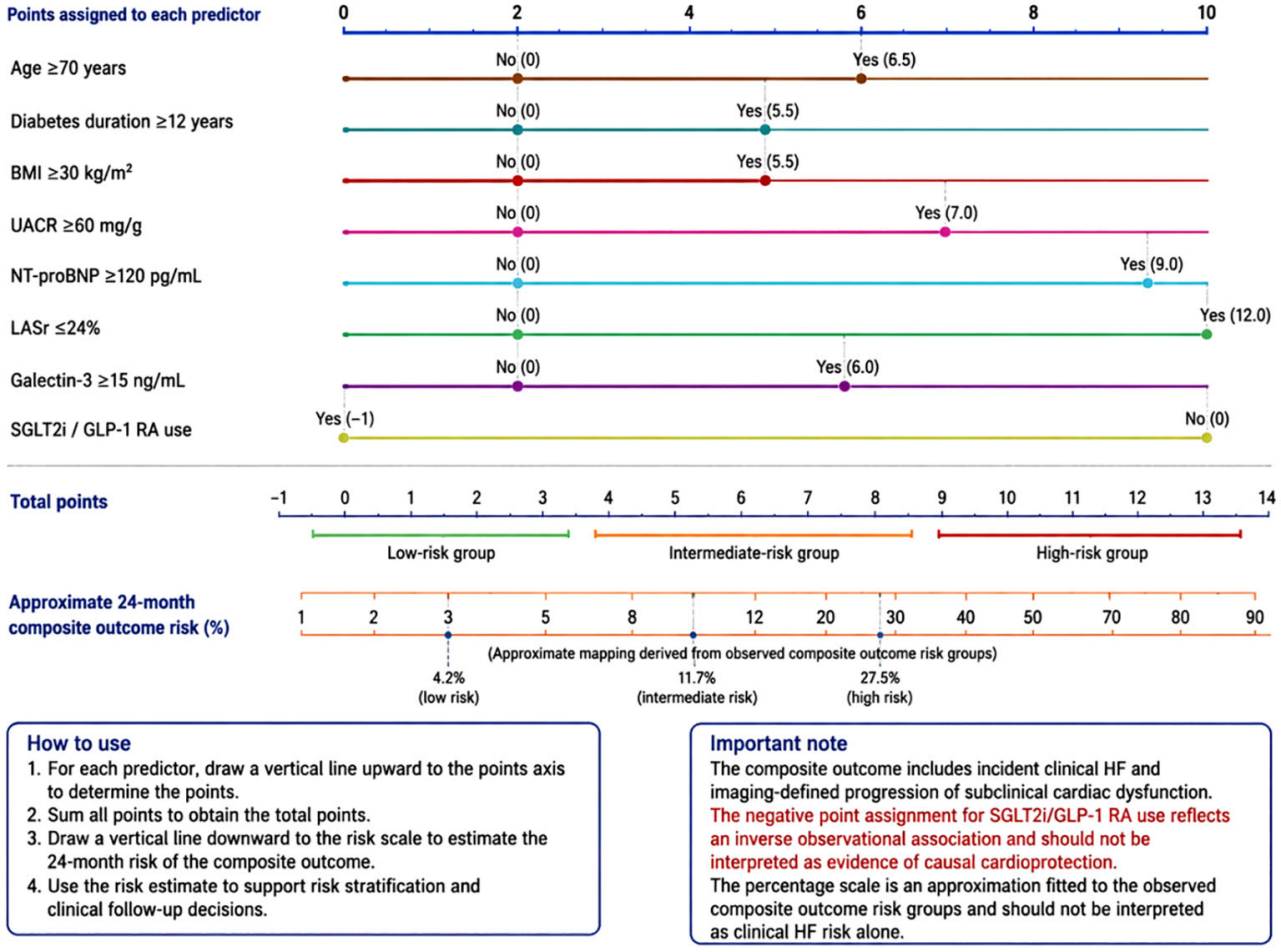
Nomogram for estimating 24-month risk of the composite outcome in asymptomatic T2DM. The composite outcome included incident clinical HF and imaging-defined progression of subclinical cardiac dysfunction. Points were assigned according to β coefficients from the final multivariable model. The negative point assignment for SGLT2i/GLP-1 RA use reflects an inverse observational association and should not be interpreted as evidence of causal cardioprotection. The estimated risk scale is approximate and requires external validation before clinical use.

LASr ≤24% showed the strongest association (HR 3.58, subdistribution HR 3.64). Other independent predictors included NT-proBNP ≥120 pg/mL, galectin-3 ≥15 ng/mL, age ≥70 years, diabetes duration ≥12 years, BMI ≥30 kg/m², and UACR ≥60 mg/g, whereas SGLT2i/GLP-1 RA use was associated with lower observed risk (non-randomized, interpret cautiously). The risk score ranged from –1 to 14 points. Bootstrap selection frequencies were 91.4% for LASr ≤24%, 86.7% for NT-proBNP, and 67.2–82.3% for other predictors. Adding LASr and galectin-3 improved the C-statistic from 0.765 to 0.835. A nomogram was constructed to estimate 24-month risk (**Figure 2**).

### 3.4 Risk stratification and model performance

**Figure 3:**
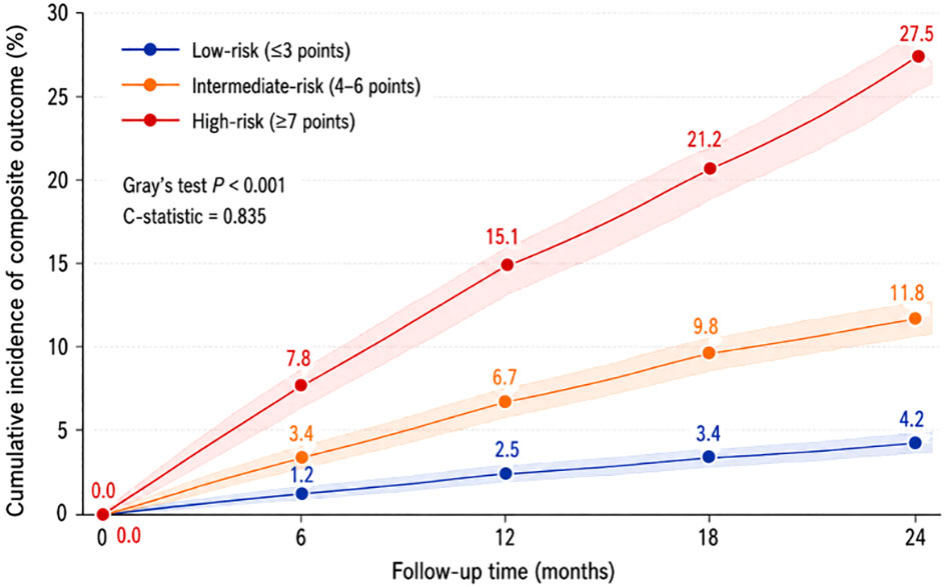
Cumulative incidence of the composite outcome by risk score group. The composite outcome included incident clinical HF and imaging-defined progression of subclinical cardiac dysfunction. Clinical HF events were further classified as HFpEF or HFrEF, whereas imaging-only progression events were analyzed separately in **Table 3**. The y-axis therefore represents the cumulative incidence of the composite outcome rather than clinical HF alone.

Based on the risk scoring system (**Table 5**), patients were stratified into low- (≤3 points), intermediate- (4–6), and high-risk (≥7) groups. The 24-month cumulative incidence of the composite outcome increased progressively across groups: 4.2%, 11.7%, and 27.5%, respectively (Gray’s test P < 0.001; **Figure 3**). Among 40 composite events, 18 were clinical HF (14 HFpEF, 4 HFrEF) and 22 were imaging-only progression, with imaging progression accounting for a substantial proportion, especially in lower-risk groups. The model demonstrated good discrimination (C-statistic 0.835) and calibration. Sensitivity analyses excluding patients with major cardiovascular interventions or stratified by SGLT2i/GLP-1 RA use showed consistent results. These findings support the potential utility of LASr, galectin-3, and clinical factors for risk stratification of incident HF or subclinical functional progression in asymptomatic T2DM.

**Table 5:** Distribution of outcome events by risk group.

Risk group	Total patients, n	Composite outcome, n (%)	Clinical HF, n (%)	HFpEF, n	HFrEF, n	Imaging-only progression, n (%)
Low-risk, ≤3 points	143	6 (4.2)	1 (0.7)	1	0	5 (3.5)
Intermediate-risk, 4–6 points	103	12 (11.7)	5 (4.9)	4	1	7 (6.8)
High-risk, ≥7 points	80	22 (27.5)	12 (15.0)	9	3	10 (12.5)
Overall	326	40 (12.3)	18 (5.5)	14	4	22 (6.7)

Clinical HF events were classified as HFpEF or HFrEF according to follow-up LVEF and clinical documentation. Imaging-only progression was defined as echocardiographic worsening without documented HF symptoms or HF hospitalization. Patients with both clinical HF and imaging progression were counted as clinical HF.

### 3.5 Model validation and performance

**Figure 4:**
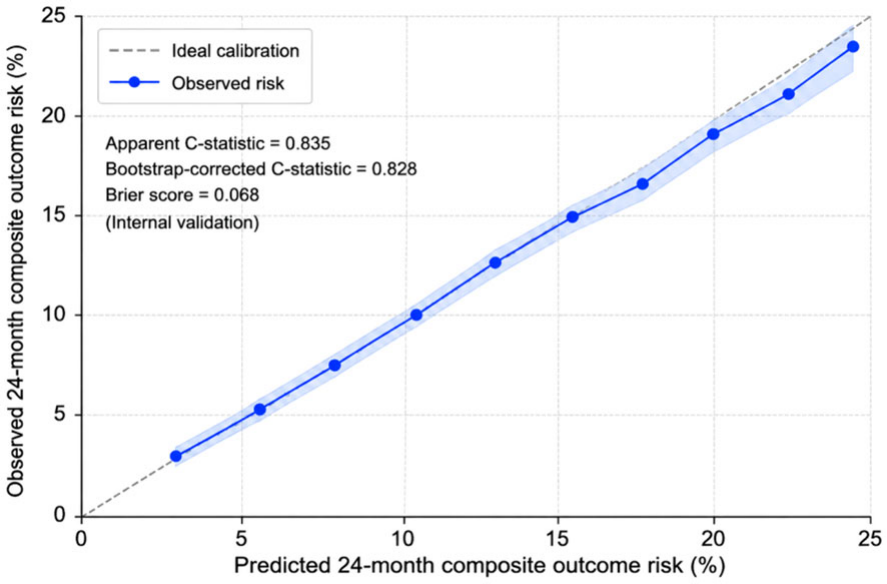
Calibration plot for 24-month composite outcome risk prediction. The x-axis shows predicted 24-month risk of the composite outcome, and the y-axis shows observed 24-month composite outcome risk.

The predictive model was internally validated using 1,000 bootstrap resamples, 5-fold cross-validation, and internal split-sample validation. The apparent C-statistic was 0.835 (95% CI 0.802–0.868), with a bootstrap-corrected value of 0.828, a mean 5-fold cross-validation C-statistic of 0.830 (range 0.823–0.837), and a split-sample validation C-statistic of 0.821. Calibration was acceptable (slope 0.93, intercept 0.02, Brier score 0.068), and the calibration plot showed good agreement between predicted and observed 24-month risk (**Figure 4**). A complete-case sensitivity analysis yielded a similar C-statistic of 0.827, indicating that results were not materially driven by imputation (**Table 6**). These findings support good discrimination and calibration of the model for the composite outcome in asymptomatic T2DM patients.

**Table 6:** Internal validation performance of the prediction model.

Validation method	C-statistic	Calibration slope	Calibration intercept	Brier score
Apparent model	0.835	0.98	0	0.068
Bootstrap-corrected model	0.828	0.93	0.02	0.07
5-fold cross-validation, mean	0.830	0.94	0.01	0.071
Internal split-sample validation	0.821	0.91	0.03	0.073
Complete-case sensitivity analysis	0.827	0.92	0.02	0.072

The internal split-sample validation used a 70:30 derivation-validation split. No external validation cohort was available. Calibration slope, calibration intercept, and Brier score were estimated at 24 months.

### 3.6 Incremental predictive value

To assess the incremental value of LASr and galectin-3, we compared three models: clinical (age, diabetes duration, BMI, UACR, NT-proBNP, medication use), clinical + LASr, and the full model (clinical + LASr + galectin-3). Adding LASr improved discrimination (ΔC = 0.058, P < 0.001) and reclassification (NRI 18.2%); adding galectin-3 yielded further modest gains (ΔC = 0.012, P = 0.02; NRI 6.5%). IDI was consistent with incremental value (**Table 7**). Exploratory decision curve analysis (5–30% risk thresholds) showed higher net benefit for the full model than the clinical-only model between ~10% and 25%, peaking at the 15% threshold (full model 0.092 vs. clinical-only 0.071). These findings suggest potential clinical utility, but external validation is required before application.

**Table 7:** Incremental predictive value of LASr and galectin-3.

Discrimination improvement			
Model	C-statistic	ΔC vs previous	P value
Clinical only	0.765	–	–
Clinical + LASr	0.823	0.058	<0.001
Clinical + LASr + Galectin-3	0.835	0.012	0.02
Reclassification improvement			
Comparison	NRI (%)	IDI	
Clinical vs Clinical + LASr	18.2	0.043	
Clinical + LASr vs Clinical + LASr + Galectin-3	6.5	0.012	

### 3.7 Subgroup and sensitivity analysis

To assess the internal robustness of the predictive model, subgroup and sensitivity analyses were conducted across clinically relevant strata: sex, age (<70 vs ≥70 years), BMI (<30 vs ≥30 kg/m²), diabetes duration (<12 vs ≥12 years), and SGLT2i/GLP-1 RA use.

The predictive performance of the model was consistent across subgroups, with C-statistics ranging from 0.820 to 0.845. LASr ≤24% and Galectin-3 ≥15 ng/mL remained independently associated with incident heart failure in all subgroups (**Table 8**). Sensitivity analyses excluding patients who underwent major cardiovascular interventions did not materially change hazard ratios or model discrimination. These results support the internal consistency of the risk model across selected clinical subgroups of asymptomatic T2DM patients, although external validation is still required to determine transportability to other populations.

**Table 8:** Subgroup analysis of model performance (C-statistic and HR for high-risk score).

Subgroup	N	HR (95% CI)	P value	C-statistic	Notes
Sex – Male	182	3.45 (2.10–5.67)	<0.001	0.832	Consistent with overall cohort
Sex – Female	144	3.52 (2.08–5.95)	<0.001	0.838	Consistent
Age <70 y	204	3.12 (1.88–5.18)	<0.001	0.828	Predictive performance maintained
Age ≥70 y	122	3.78 (2.15–6.63)	<0.001	0.842	Slightly higher risk in older adults
BMI <30 kg/m²	261	3.33 (2.03–5.46)	<0.001	0.83	Model stable across BMI
BMI ≥30 kg/m²	65	3.68 (1.88–7.21)	<0.001	0.84	Limited sample but consistent
Diabetes duration <12 y	196	3.21 (1.95–5.28)	<0.001	0.826	Model valid in shorter disease duration
Diabetes duration ≥12 y	130	3.74 (2.12–6.61)	<0.001	0.839	Model valid in longer disease duration
SGLT2i/GLP-1 RA use	107	3.12 (1.78–5.46)	<0.001	0.827	Effect independent of therapy
Non-use	219	3.51 (2.20–5.60)	<0.001	0.836	Model robust

## 4 Discussion

DCM is a diabetes-related myocardial phenotype characterized by subclinical cardiac dysfunction, structural remodeling, and increased risk of subsequent HF in patients with T2DM. However, in clinical cohorts, this phenotype is often heterogeneous and may substantially overlap with ACC/AHA Stage B HF, hypertension-related remodeling, obesity-associated cardiomyopathy, aging-related myocardial changes, or occult ischemic disease. Evidence regarding long-term interventions targeting this evolutionary process remains limited. In this context, our model should be interpreted as a tool for identifying asymptomatic T2DM patients at increased risk of incident HF or progression of subclinical cardiac dysfunction, rather than as a diagnostic model for DCM-specific pathology (17).

The predictors retained in our model reflect distinct biological dimensions of early diabetic myocardial injury. Reduced LASr (≤24%) may indicate impaired left atrial compliance and chronic diastolic burden, which are early consequences of increased myocardial stiffness. Abnormal GLS captures early longitudinal systolic impairment that typically precedes a decline in LVEF. Elevated NT-proBNP (≥120 pg/mL) reflects increased myocardial wall stress secondary to subclinical diastolic and systolic dysfunction. Galectin-3 (≥15 ng/mL) may represent fibrotic and inflammatory activity, as this lectin is involved in cardiac fibroblast activation and extracellular matrix remodeling. These associations are biologically plausible, given that diabetic myocardial remodeling is closely linked to metabolic stress, oxidative injury, inflammation, mitochondrial dysfunction, and extracellular matrix remodeling.

Although the present study did not directly measure molecular pathways, recent multi-omics studies provide complementary mechanistic context. Transcriptomic analyses have suggested that altered inflammatory and metabolic gene programs may participate in diabetic myocardial remodeling (18, 19). Proteomic profiling may help identify circulating or myocardial proteins related to fibrosis and metabolic stress (20). Metabolomic studies indicate that disturbed substrate utilization and metabolic rewiring are closely related to cardiac dysfunction in diabetes (21). Epigenetic biomarkers may provide another layer of regulation linking chronic metabolic exposure to myocardial remodeling (22).

Our longitudinal findings are consistent with and extend prior work. In our cohort, patients exhibiting slower worsening trajectories showed only modest declines in LV GLS (from 17.4 ± 2.0% to 16.9 ± 2.1%), minimal E/e′ elevation (11.3 ± 2.6 to 12.0 ± 2.8), and slight increases in LAVI (35.0 ± 6.5 mL/m² to 36.2 ± 6.8 mL/m²) over 24 months, indicating preserved myocardial electromechanical coupling and structural integrity (23, 24). These trends align with studies demonstrating that alterations in myocardial strain predict subsequent ventricular remodeling and adverse outcomes in asymptomatic T2DM. Specifically, Ernande et al. (2014) showed that longitudinal myocardial strain alteration is associated with left ventricular remodeling (23), and Jørgensen et al. (2016) demonstrated that cholesterol remnants and triglycerides are linked to decreased myocardial function (24). Bojer et al. (2024) reported that early signs of myocardial systolic dysfunction are strongly associated with myocardial microvascular dysfunction independent of fibrosis (25). In a follow-up study, Bojer et al. (2023) found that myocardial extracellular volume and blood flow independently affect cardiac diastolic function (26), which aligns with our observation that changes in E/e′ and LAVI occurred prior to significant LVEF reduction. Jørgensen et al. (2019) highlighted the predictive value of echocardiography in T2DM (27), and Tanaka et al. (2020) demonstrated that diabetes exacerbates left ventricular longitudinal function impairment in non-ischemic dilated cardiomyopathy (28). Our subgroup analyses showed that patients with higher baseline LV GLS and lower NT-proBNP had slower deterioration of strain parameters, further supporting the predictive utility of these indices.

Moreover, composite indices like MMEI, SFCI, and DBI, though exploratory, complemented the primary metrics by integrating mechanical efficiency with structural load. In our cohort, MMEI declined modestly from baseline to 24 months in patients with slower progression, whereas SFCI and DBI values remained relatively stable, providing additional insight into subclinical electromechanical stability (29, 30). The differential evolution of LV GLS and E/e′ was evident early during follow-up, preceding overt changes in LVEF, suggesting that early subclinical impairment can be captured and quantified. This is consistent with mechanistic evidence that microvascular dysfunction and myocardial extracellular volume fraction influence early diastolic relaxation independently of fibrosis (31, 32).

Medication-related findings should be interpreted cautiously. In the multivariable model, SGLT2i/GLP-1 RA use was associated with lower observed risk of the composite outcome, and treated patients showed numerically smaller deterioration in LV GLS, E/e′, and LAVI during follow-up. However, these findings should not be interpreted as evidence of causal cardioprotection. Treatment allocation was not randomized, and medication use may reflect differences in disease severity, physician preference, access to care, metabolic control, renal status, and overall quality of risk-factor management. Although prior interventional and observational studies have reported favorable effects of GLP-1 receptor agonists and SGLT2 inhibitors on cardiac structure, strain indices, NT-proBNP, or clinical HF outcomes (33–35), the present study was not designed to estimate treatment efficacy. Therefore, medication use was retained in the model primarily as a clinical covariate rather than as a causal therapeutic predictor. In a sensitivity analysis using propensity score adjustment for age, sex, diabetes duration, BMI, HbA1c, eGFR, UACR, hypertension, CKD, baseline NT-proBNP, baseline GLS, and baseline LASr, the association between SGLT2i/GLP-1 RA use and the composite outcome was attenuated and no longer statistically significant (adjusted HR 0.68, 95% CI 0.42–1.11; P = 0.12). This attenuation supports the possibility that the apparent protective association may partly reflect confounding by indication or differences in baseline risk management.

The trajectory analyses in our study highlight the value of repeated measurements and mixed-effects modeling in detecting subtle changes before conventional endpoints. By explicitly accounting for within-subject correlation, these models provided statistical evidence of progressive changes in LASr, LV GLS, E/e′, and LAVI over 24 months. In addition, the good-to-excellent inter-observer and intra-observer reproducibility of LASr and LV GLS (ICCs 0.88–0.94) supports the measurement reliability of these strain-derived predictors. Nevertheless, measurement reliability and improved discrimination do not necessarily translate into clinical benefit. The model should therefore be viewed as a preliminary risk stratification tool that requires external validation, decision-curve confirmation, and prospective testing before routine clinical use.

Several limitations warrant consideration. First, single-center retrospective design and lack of external validation limit generalizability. Second, modest sample size (40 events) and evaluation of 26 candidates may introduce instability; backward elimination was exploratory. Third, MICE under MAR assumption cannot be fully verified. Fourth, residual confounding (lifestyle, hypertension severity, medication adherence, etc.) may persist; SGLT2i/GLP-1 RA association is not causal. Fifth, echocardiography cannot fully differentiate DCM from mixed phenotypes or capture tissue-level fibrosis/microvascular dysfunction. Sixth, 24-month follow-up may be insufficient, and the composite endpoint includes both clinical HF and imaging-only progression, which differ in severity. Seventh, exploratory indices (MMEI, SFCI, DBI) lack external validation. Finally, clinical implementation requires external validation, decision-curve analysis, cost-effectiveness assessment, and prospective testing.

## 5 Conclusion

This study developed an internally validated prediction framework for incident HF or subclinical progression in asymptomatic T2DM patients with subclinical myocardial abnormalities. Repeated 24-month measurements showed that early markers (LV GLS, E/e′, LAVI) and composite indices can detect differential progression trajectories, enabling refined risk stratification. Integrating sensitive echocardiographic indices, biomarkers, and clinical parameters may improve statistical risk prediction and preliminary stratification in high-risk T2DM patients. Future multicenter prospective studies with independent external validation are needed to confirm model transportability, recalibrate risk estimates, evaluate clinical utility, and determine whether model-guided stratification improves individualized management or outcomes.

## Data Availability

The raw data supporting the conclusions of this article will be made available by the authors, without undue reservation.
